# Public health round-up

**DOI:** 10.2471/BLT.16.010716

**Published:** 2016-07-01

**Yellow fever vaccination in DRC after Angola outbreak**

Children in the province of Kongo Central in the Democratic Republic of the Congo wait to be vaccinated against yellow fever. More than 1.9 million people in the country have been vaccinated against the mosquito-borne disease since 26 May. The campaign started after cases were imported from neighbouring Angola, where efforts to quell the country’s worst yellow fever outbreak in 30 years continued last month.

http://www.who.int/emergencies/yellow-fever/situation-reports/2-june-2016/en/

**Figure:**
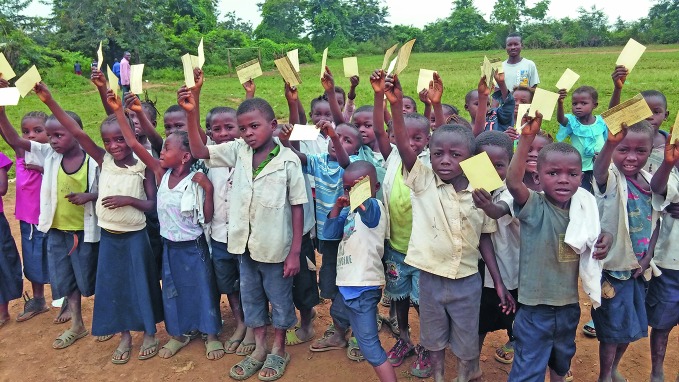
WHO/ Honoré Mbaya

## Zika and sexual transmission

Governments in countries where Zika virus is circulating should ensure that people who are infected, and their sexual partners, are informed about the risks of sexual transmission of the virus and provided with condoms according to updated WHO guidance.

Zika virus has recently become associated with adverse foetal outcomes, including microcephaly, as well as neurological complications and Guillain-Barré syndrome. The virus is primarily transmitted by the bite of the *Aedes aegyptii* mosquito, but there is increasing evidence of sexual transmission.

In the updated guidance, WHO recommended that countries where the virus is circulating should ensure that women who have had unprotected sex, and do not wish to become pregnant due to concerns about Zika virus infection, have ready access to emergency contraceptive services and counselling.

Public health authorities in these countries should ensure that men and women of reproductive age receive counselling and evidence-based information to allow them to make informed decisions about any plans for pregnancy. 

In addition, the sexual partners of pregnant women, living in or returning from areas where local transmission of Zika virus has been established, should practice safe sex or abstinence during the pregnancy.

Couples or women planning a pregnancy returning from areas where Zika virus is circulating are recommended to wait at least eight weeks before trying to conceive, to ensure that any possible Zika virus infection has cleared, and 6 months, if the male partner was symptomatic.

The guidance is based on 12 studies and reports that have been published since 2011.

All refer to sexual transmission of Zika by symptomatic men. There are no reports of sexual transmission of Zika by women or asymptomatic men.

WHO does not recommend routine semen testing to detect Zika virus. However, symptomatic men can be offered semen testing at the end of an eight-week period after their return according to the country’s policy, the guidance said.

http://who.int/csr/resources/publications/zika/sexual-transmission-prevention

## WHO reports attacks on health care

Some 594 attacks on health care were reported between January 2014 and December 2015 resulting in 959 deaths and 1561 injuries in 19 countries facing emergencies, according to a new WHO report.

More than half of the attacks (377) targeted health-care facilities and nearly a quarter (156) of them targeted health-care workers, according to WHO’s *Report on attacks on health care in emergencies*.

In 2015, an estimated 125 million people affected by emergencies were in need of humanitarian assistance – the largest number ever on record, according to the United Nations.

Currently there is no publicly available source of consolidated information on attacks on health care in countries facing emergencies. 

The WHO report that was released at the end of May is an attempt to consolidate and analyse the data that is available from open sources.

The largest share of these attacks during the two-year period were in the Syrian Arab Republic (228), followed by the West Bank and Gaza Strip (53), Iraq (43), Pakistan (43) and Libya (33).

“Such attacks not only endanger health-care providers, they also deprive people of urgently needed care when they need it most,” the report said.

“And while the consequences of such attacks are as yet largely undocumented, they are presumed to be significant – negatively affecting short-term health-care delivery as well as the longer-term health and well-being of affected populations.”

http://www.who.int/hac/techguidance/attacks_on_health_care

## HIV and breastfeeding

Mothers living with HIV should breastfeed their babies for at least 12 months and can continue for up to 24 months or longer (as for the general population) while being fully supported for antiretroviral treatment including adherence support, according to recently released WHO guidance. This recommendation is for countries where national authorities already promote and support breastfeeding among mothers living with HIV.

The guidance, entitled *Updates on HIV and infant feeding*, is an update of recommendations issued in 2010.

It calls on national and local health authorities to actively provide services in health facilities and coordinate activities in workplaces, communities and homes that protect, promote and support breastfeeding among women with HIV.

“Mothers living with HIV and health-care workers can be reassured that antiretroviral treatment is effective at reducing the risk of postnatal HIV transmission in the context of mixed feeding and that mixed feeding in itself is not a reason to stop breastfeeding,” the guidance says.

When mothers with HIV infection do not plan to breastfeed for 12 months because they want to return to work or school, then a shorter duration of planned breastfeeding while they are on antiretroviral treatment is still better than no breastfeeding at all, according to the guidance.

Exclusive breastfeeding for 6 months has many benefits for both the infant and mother. In particular, breastfeeding reduces the risk of infectious disease and improves early child development. It also reduces the risk of breast cancer among women.

In addition, initiation of breastfeeding within one hour of birth protects the newborn from acquiring infections and reduces mortality in this critical early period. The risk of death due to diarrhoeal diseases and other infections can increase in infants who are either partially breastfed or not breastfed at all.

However, many infants and children do not receive optimal feeding. Worldwide, only an estimated 36% of all infants were exclusively breastfed until they were 6 months old during 2007–2014.

## Health and climate meeting in Paris

Government officials, scientists and civil society representatives gather this month in Paris to discuss their countries’ plans to protect human health from the ill-effects of climate change.

The meeting follows up on commitments made by countries at the United Nations climate change conference in Paris last year to curb greenhouse gas emissions in order to keep global warming below 2°C this century.

Countries agreed to prepare and implement plans to protect human health from the worst effects of climate change, such as air pollution, heat waves, floods and droughts, as well as the decline of water resources and food security.

More than 7 million deaths worldwide are attributed to air pollution every year.

The Second Global Conference on Health and Climate takes place in Paris from 7–8 July and is jointly hosted by the French government and WHO.

WHO is appointing a working group to prepare a report on the economic costs of the disease burden due to climate change and fossil-fuel use, and on the co-benefits of replacing fossil fuels – coal, oil, gas and petroleum – with clean and renewable sources of energy. 

It is expected to report its findings to the World Health Assembly in May 2017.

http://www.who.int/globalchange/mediacentre/events/climate-health-conference

**Cover photo**

This month’s cover photograph shows a Himalayan glacier in the Zanskar valley in northern India that is shrinking. Global warming is altering the earth’s climate system, including its land, atmosphere and oceans and reducing ice and snow in mountainous areas.

**Figure:**
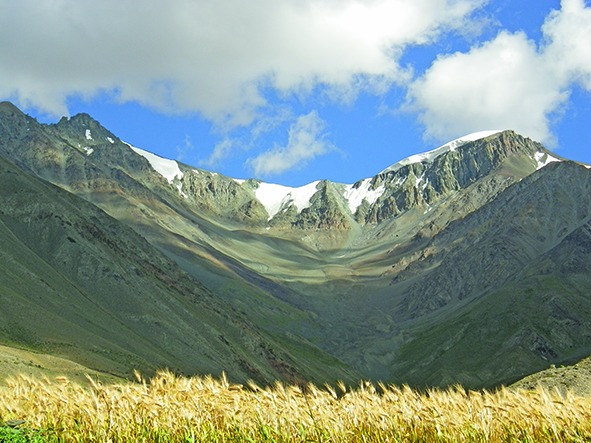
Courtesy of Jonathan Mingle

## STI guidelines address antimicrobial resistance

WHO urged countries to update their treatment protocols for sexually-transmitted infections (STI) in response to growing antimicrobial resistance (AMR).

New approaches are urgently needed and possible, given recent advancements in the prevention, diagnosis and treatment of these infections, it said. Effective treatment protocols that take account of global and local resistance patterns are essential to reduce the further development of AMR.

Last month WHO released the first two of several planned sets of treatment guidance on STIs for *Neisseria gonorrhoeae* and *Chlamydia trachomatis.* The full set of guidelines on STIs, which updates those from 2003, will be issued in phases over the next couple of years.

*N. gonorrhoeae*, the bacterium that causes gonorrhoea, has developed high-level resistance to first-line medicines (called quinolones) to treat this infection, and resistance to cephalosporins, another first-line treatment, is also emerging the guideline said.

Quinolones are no longer recommended for the treatment of gonorrhoea due to the reported high-level of resistance. In places, where local resistance data are not available, the new guideline suggests treatment with both ceftriaxone and azithromycin.

For *C. trachomatis*, the most common bacterial STI, azithromycin and doxycycline remain the treatment of choice for uncomplicated genital and anorectal infections.

There are few reported treatment failures for chlamydia for two groups of medicines, called tetracyclines and macrolides. The potential for resistance to azithromycin, doxycycline and other treatment options should be investigated further, the new guideline said.

In addition to these two new guidelines, WHO is developing guidelines on *Treponema pallidum* (the spirochete that causes syphilis) and herpes simplex virus.

Other guidelines will be on the screening for and treatment of syphilis in pregnant women; on STI syndromic approach (diagnosis based on the identification of a consistent group of symptoms and easily recognized signs and treatment based on the majority of organisms causing the syndrome); on the clinical management package including partner notification, prevention and treatment of other STIs; and on STI laboratory diagnosis and screening.

**Looking ahead**

28 July – World Hepatitis Day

19 September – United Nations Summit on Refugees and Migrants

14–20 November – World Antibiotic Awareness Week

1 December – World AIDS Day

